# Actinomyces odontolyticus: A Rare Agent of Exit-Site Infection in Peritoneal Dialysis

**DOI:** 10.7759/cureus.76273

**Published:** 2024-12-23

**Authors:** Mariana Freitas, Bárbara Beirão Rodrigues, Luís Oliveira, Rui Castro, Teresa Morgado

**Affiliations:** 1 Nephrology, Centro Hospitalar de Trás-os-Montes e Alto Douro, Vila Real, PRT

**Keywords:** chronic kidney disease (ckd), continuous ambulatory peritoneal dialysis, infections in peritoneal dialysis, peritoneal dialysis complication, peritoneal dialysis (pd)

## Abstract

Exit-site infections (ESIs) of peritoneal dialysis catheters can cause serious complications if not promptly treated. Uncommon pathogens like *Actinomyces odontolyticus* are infrequently associated with these infections. We report a 26-year-old woman with end-stage renal disease due to Alport syndrome, presenting with recurrent purulent discharge and erythema at the Tenckhoff catheter exit site. Initial cultures were negative, and empirical treatment with cotrimoxazole was provided with an apparent resolution of the infection. Upon recurrence, *A. odontolyticus* was identified in an anaerobic culture, showing resistance to multiple antibiotics, including penicillin. Treatment with doxycycline resolved the infection within four weeks without requiring catheter removal. No recurrence was observed after six months. This case highlights the importance of early diagnosis and tailored antibiotic therapy in managing rare infections. It emphasizes the need for collaboration with microbiology labs to ensure proper identification of atypical pathogens. Early intervention and targeted treatment can lead to excellent outcomes, potentially avoiding catheter removal.

## Introduction

Peritoneal dialysis (PD) catheter-related infections, such as exit-site and tunnel infections, can result in significant morbidity if not promptly identified and effectively treated. Exit-site infections (ESIs) can progress to tunnel infections and/or peritonitis, and may sometimes require hospitalization or even transfer to hemodialysis [1]. Early diagnosis and appropriate treatment are crucial to prevent these adverse outcomes. The most frequently reported pathogens associated with PD catheter ESIs include *Staphylococcus aureus* and *Pseudomonas aeruginosa *[2]. Rarely, atypical organisms such as *Actinomyces odontolyticus* have been identified. As far as we know, only one case of ESI due to *A. odontolyticus* in a patient on continuous ambulatory peritoneal dialysis (CAPD) has been reported [3], highlighting the exceptional rarity of this pathogen in the context of ESI.

*A. odontolyticus* is an anaerobic gram-positive bacteria, which can be found among the commensal flora of the mouth and oropharynx [4]. Diagnosis of infections due to this microorganism can be difficult because of its rarity, indolent course, and lack of specific symptoms [3].

This clinical report aims to describe a rare case of *A. odontolyticus* ESI in a PD patient, detailing the clinical presentation, diagnostic challenges, treatment, and prognosis.

This case report was presented as an e-poster at the 60^th^ European Renal Association Congress in Milan, June 15-18, 2023.

## Case presentation

**Figure 1:**
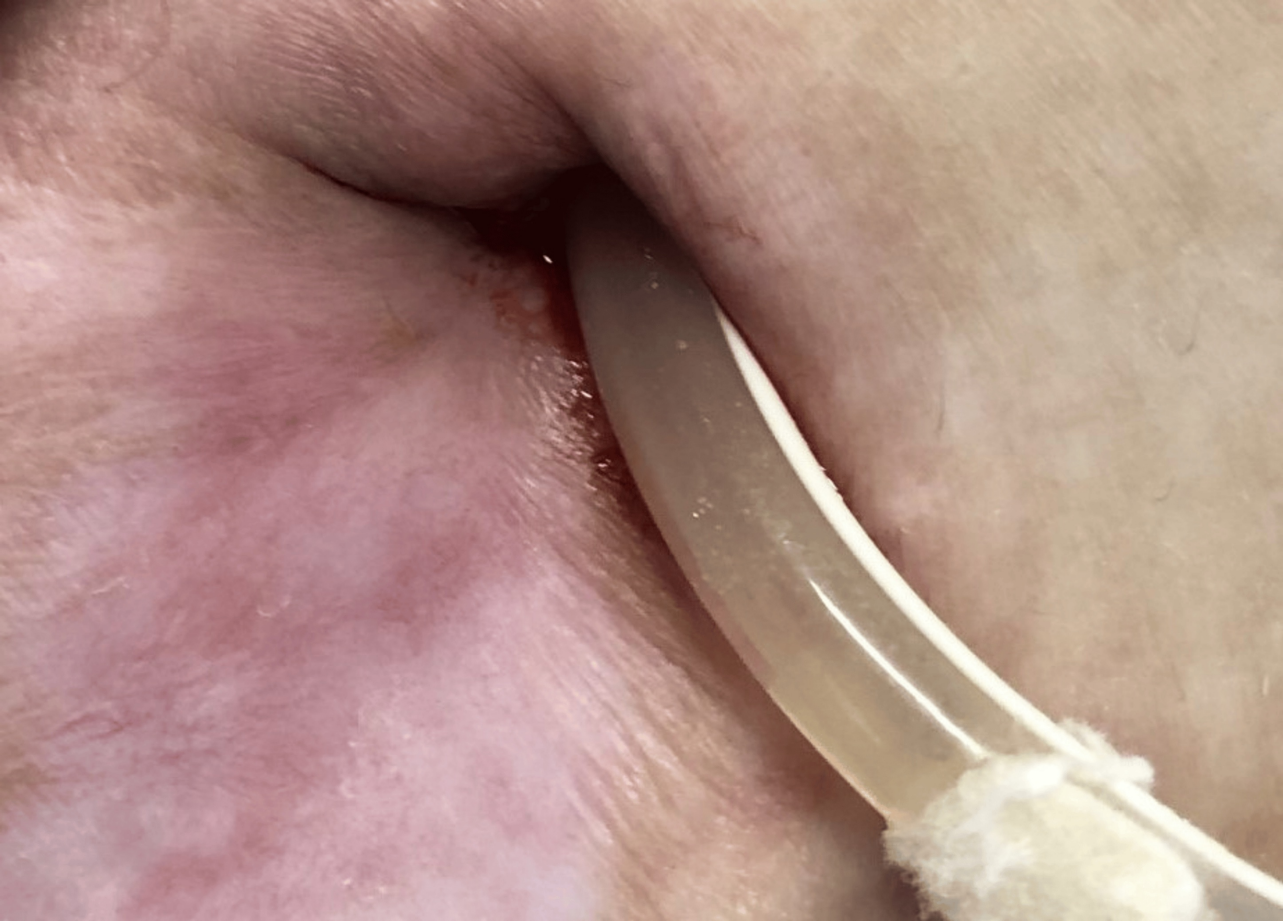
Exit site of the Tenckhoff catheter with erythema of the skin and purulent discharge.

We present a case of a 26-year-old woman with a medical history of end-stage chronic kidney disease (CKD) secondary to Alport syndrome, dyslipidemia, and arterial hypertension. She has been on continuous ambulatory peritoneal dialysis (CAPD) since April 2016. In June 2020, the patient presented an episode of refractory ESI due to methicillin-sensitive *Staphylococcus aureus* and was submitted to shaving of the external cuff of the Tenckhoff catheter, with favorable subsequent evolution. The patient had no more complications related to the catheter until April 2021, when she presented purulent drainage through the exit site associated with erythema in the surrounding area. Exit-site swabs were negative. The patient was on antibiotic prophylaxis with topical mupirocin, commonly used to prevent infections caused by *Staphylococcus aureus*. Due to concerns about resistance from prolonged use and to expand the antimicrobial spectrum to include gram-negative coverage, mupirocin was switched to topical gentamicin. Also, a two-week course of oral cotrimoxazole was initiated to provide systemic coverage against both gram-positive and gram-negative bacteria, guided by the microbiological chart of our unit. This decision reflected local antibiograms and pathogen susceptibility patterns, ensuring a targeted antimicrobial strategy tailored to the patient’s clinical history and infection risk. The ESI seemed to resolve with these measures; however, in May 2021, erythema and purulent drainage recurred (Figure 1), and a rare pathogen, *Actinomyces odontolyticus*, was identified in the swab.

The strain exhibited resistance to penicillin, amoxicillin, ceftriaxone, and cotrimoxazole. On the other hand, it showed sensitivity to doxycycline, clindamycin, and carbapenems. Based on susceptibility testing, the patient was treated exclusively with doxycycline 100 mg every 12 hours for four weeks. No collections were found in the ultrasound assessments of the catheter tunnel at the time of diagnosis and during follow-up evaluations. The evolution was favorable with the resolution of the infection and the absence of recurrence for six months. After that, the patient received a kidney transplant.

## Discussion

*Actinomyces odontolyticus* is a gram-positive, anaerobic bacterium of the commensal flora of the oral cavity, upper gastrointestinal tract, and female genital tract [4,5]. These bacteria seem to acquire pathogenicity in situations of impaired mucosal integrity, such as surgery or trauma, and in immunosuppressed patients [4]. Infections caused by this organism often manifest as granulomatous and suppurative lesions, with abscess formation being a hallmark of actinomycosis. The disease generally remains localized; however, it may spread to contiguous structures, while hematogenous dissemination is uncommon [6]. In humans, the cervicofacial region is the most commonly affected by this agent; nevertheless, *A. odontolyticus* has also been implicated in infections of the respiratory tract, abdominal cavity, and pelvic organs [4]. Uncommonly, it can cause bacteremia, particularly in immunocompromised patients or carriers of medical devices [7-9]. Although abdominal actinomycosis accounts for 10%-20% of the described cases, peritoneal involvement is particularly infrequent and rarely reported in PD patients [3,5,10,11].

Tenckhoff catheter ESI caused by *A. odontolyticus* is extremely rare, with the first case described by Siu et al. in 2004 [3]. To our knowledge, the present case report represents the second ESI attributed to this organism. In Siu et al.'s [3] case report, the patient had repeated episodes of ESI, which were considered to be the predisposing factor for infection by this opportunistic pathogen. The authors detailed an initial ESI that subsequently progressed to micro-abscess formation in the surrounding area. Our patient had a prior history of external cuff shaving of the Tenckhoff catheter due to a refractory infection caused by *Staphylococcus aureus*. This prior intervention, by compromising skin integrity, may have facilitated the occurrence of ESI by this pathogen. Furthermore, it is important to note that the other episode of ESI that our patient presented in the previous month, which did not have an identified agent, was very likely caused by *A. odontolyticus*. The failure to isolate the organism at that time may have been due to insufficient culture time for the growth of the agent. Indeed, the diagnosis of infections by *A. odontolyticus* is challenging not only because of its rarity but also due to its growth in an anaerobic environment and prolonged isolation time, ranging from five to seven days to four weeks [11]. In the case described by Siu et al. [3], the pathogen could not be isolated despite several attempts, until anaerobic culture methods were included. This highlights the importance of close collaboration with microbiology departments. In cases without pathogen isolation or when there is a clinical suspicion of atypical agents, adjustments to culture media and incubation times should be reappraised to ensure proper identification. In our case, the use of topical antibiotic prophylaxis and prior treatment with cotrimoxazole may explain the absence of simultaneous isolation of other more common ESI-causing agents, such as *Staphylococcus aureus*. Being resistant to these antibiotics, *A. odontolyticus* was able to proliferate and dominate the local microenvironment.

There are no randomized controlled trials assessing the efficacy of antimicrobial regimens for the treatment of actinomycosis [12]. Penicillin is considered the first-line treatment for most actinomycosis, including those caused by *A. odontolyticus*. High doses should be administered for an extended period, often four to six weeks, followed by oral therapy with amoxicillin to ensure complete eradication [3,12,13]. There is no consensus on the total duration of the treatment; however, some authors suggested that therapy should be extended for at least one to two months after the infection has resolved, typically ranging from two to six months for mild cases and six to 12 months for more severe cases [3,12]. Alternative antibiotic regimens such as ceftriaxone, doxycycline, clindamycin, or carbapenems may be used in cases of penicillin allergy or resistance [12]. Thus, antibiotic susceptibility testing is crucial, as variations in resistance patterns require personalized treatment approaches for each patient. Surgical intervention, involving abscess drainage or infected tissue removal, is sometimes required in association with antimicrobial therapy to achieve optimal outcomes. In the case described by Siu et al. [3], the patient presented signs of ESI and posteriorly developed small collections along the catheter tunnel tract, down to the distal cuff. The authors reported treatment with intravenous penicillin G at a dose of 2 MU every six hours for four weeks, followed by six months of oral amoxicillin. They did not remove the peritoneal dialysis catheter and adopted a strategy of close clinical and imaging monitoring, reporting that the exit site began to dry up after one month of intravenous penicillin and cleared entirely during the second month of treatment with oral amoxicillin. The patient then remained symptom-free for over a year with no recurrence. In fact, there is no consensus on whether catheter removal is necessary in such cases due to a lack of data. In our patient, there was no evidence of collections in the surrounding area, which may suggest that the diagnosis was made earlier before complications developed. Based on the antibiotic susceptibility testing that we had access to, treatment with doxycycline 100 mg every 12 hours was initiated. Doxycycline was chosen for its excellent oral bioavailability, broad tissue penetration, and convenience for outpatient management. The patient showed significant improvement after one week of therapy and evolved without collections. She completed four weeks of treatment, achieving infection resolution without requiring catheter removal. As previously mentioned, the treatment duration for infections caused by this agent is not consensual. Our choice of a four-week course was based on the recommendations of some authors [3,12], suggesting a treatment duration of one to two months following infection resolution. Taking into account the patient’s significant clinical improvement observed during the first week of treatment, we considered that a shorter course of four weeks was sufficient to achieve effective resolution while reducing the risk of unnecessary prolonged antibiotic use. The patient remained asymptomatic for six months, afterwards she underwent a kidney transplant. These two cases illustrate that when a positive response to medical treatment occurs within the initial weeks, the infection can be effectively managed without requiring catheter removal. In their work, Siu et al. [3] postulate that if signs of infection worsen after two weeks of intravenous therapy or no improvement is observed after one month, catheter removal and drainage of collections should be considered. Our experience with our case highlights the value of early diagnosis and demonstrates that *A. odontolyticus* ESI of the PD catheter can achieve excellent outcomes with a relatively short course of treatment.

## Conclusions

This case report contributes to the limited literature on* Actinomyces odontolyticus* ESI of the Tenckhoff catheter in patients under CAPD. Our findings, along with the previously reported case, highlight the importance of early diagnosis and appropriate antibiotic therapy in managing this rare infection. Early intervention appears to significantly improve outcomes and may eliminate the need for catheter removal, as seen in our case. Close collaboration with microbiology departments is essential to ensure proper pathogen identification, especially given the organism’s slow growth.
